# Ditangquan exercises based on safe-landing strategies prevent falls and injury among older individuals with sarcopenia

**DOI:** 10.3389/fmed.2022.936314

**Published:** 2022-08-16

**Authors:** Zhen-rui Li, Yun-jing Ma, Jie Zhuang, Xun-chen Tao, Chao-yang Guo, Shu-ting Liu, Ran-ran Zhu, Jin-xiang Wang, Lei Fang

**Affiliations:** ^1^Yueyang Hospital of Integrated Traditional Chinese and Western Medicine, Shanghai University of Traditional Chinese Medicine, Shanghai, China; ^2^Department of Rehabilitation, Shanghai East Hospital, Shanghai, China; ^3^School of Rehabilitation Science, Shanghai University of Traditional Chinese Medicine, Shanghai, China

**Keywords:** sarcopenia, safe landing, modified falls efficacy scale, traditional Chinese exercise, fall injury prevention

## Abstract

**Background:**

Sarcopenia is the age-related loss of skeletal muscle mass and function; it is a risk factor for falls among older individuals. Few studies have focused on training such individuals to adopt a safe-landing strategy that would protect them from fall-related injuries. Ditangquan is a traditional Chinese martial art comprising movements that conform to the principles of safe landing. This study aims to investigate the effectiveness of Ditangquan in preventing fall-related injuries among older individuals with sarcopenia.

**Methods:**

A total of 70 participants (21 males and 49 females with sarcopenia) between 60 and 80 years of age were recruited from three local communities and randomly assigned to the Ditangquan exercise group (DG) or the control group (CG) in a 1:1 ratio. Three times a week for 24 weeks, both the DG and CG received an hour of conventional exercise and an hour of Ditangquan exercise based on safe landing. Primary outcomes were the modified falls efficacy scale (MFES), the number of falls, and fall injuries; the secondary outcome was the Timed Up & Go (TUG) test.

**Results:**

The DG had significantly fewer falls (1 vs. 8, *P* = 0.028) and fall injuries (0 vs. 6, *P* = 0.025) than the CG. Furthermore, at the end of the study, the DG had a significantly improved MFES (mean difference: 32.17 scores; 95% CI: 21.32, 43.02; *P* <0.001) and TUGT (mean difference: −4.94 s; 95% CI: −7.95, −1.93; *P* = 0.002) as compared with the CG.

**Conclusion:**

Ditangquan exercise based on the safe-landing strategy effectively improves the functional mobility of the elderly, reduces the occurrence of falls and injuries, and increases the individual's confidence in preventing falls.

## Introduction

In 2021, a branch of the Chinese Medical Association published a consensus report (1, 2) that defined skeletal muscle reduction (sarcopenia) as a clinical syndrome characterized by decreased skeletal muscle mass and/or muscle strength or decreased physiologic function of the muscles as related to the aging process. Sarcopenia is likely to increase the incidence of osteoporosis and fractures among older adults (3).

More than 17% of seniors in mainland China suffer from sarcopenia (4), and it is estimated that as many as 200 million will develop this condition over the next 40 years (5). Sarcopenia is one of the main physical factors that lead to falling among the aged (6), and such falls commonly lead to disability and death (7–9). Sarcopenia severely affects the physical and psychological health of the aged, placing a heavy personal and financial burden on their families as well as on society (10, 11). Most preventive guidelines recommend resistance exercise as the most effective way to build muscle mass and strength, but falls continued to occur among those who have undergone such treatment (12–14).

In our previous study on the biomechanics of the Ditangquan exercise (15), we found that Ditangquan movements can significantly decrease the nearly 1,000-N (newton) force of impact when a person's body falls to the floor, thereby minimizing physical injury. We therefore found it necessary to design an exercise program for older adults that would incorporate safe-landing strategies and would thus protect the individual from being injured.

## Methods

### Study design

This study was designed as a parallel-group, analyst-blinded, randomized controlled trial (RCT) conducted at three different institutions: the Shanghai Huangpu District Nanchang Road No. 44 Elder Care Home, Xuhui District Dapuqiao Road Elder Care Home, and Pudong New District Shendong hospital in Shanghai between June 2019 and December 2021. All eligible participants signed an informed consent form after confirming their willingness to participate. They were randomly assigned to the Ditangquan exercise group (DG) and the control group (CG) in a 1:1 ratio. The study protocol was conducted in accordance with the Declaration of Helsinki and was approved by the Ethics Committee of Yueyang Hospital of Integrated Traditional Chinese and Western Medicine (No. 2018-013). We registered the study on the Chinese Clinical Trial Registry (No. ChiCTR1800016562).

### Participant recruitment

We recruited some elderly participants with histories of falls by offering health lessons in the community and publicizing our work through posters, internet advertisements, and WeChat platforms.

#### Inclusion criteria

Each prospective participant had to (1) have a history of falling in the past 2 years, and 15.96 s was used as a cutoff point of the Timed Up & Go (TUG) test to screen for recurrent falls (16); (2) be willing to be randomly assigned to receive intervention for 24 weeks, and sign informed consent; (3) meet the diagnostic criteria for sarcopenia (17) without gender limit for participants aged 60 to 80 years; (4) agree not to engage in other forms of exercise during the study period; (5) have a body mass index (BMI) between 18 and 25 kg/m^2^; and (6) be alert and able to walk independently or with the help of aid such as a cane.

#### Exclusion criteria

Participants having any of the following conditions were excluded: (1) chronic metabolic disorders, serious cardiovascular disease, hypertension, and/or obesity; (2) mental illness; (3) recent muscle, joint, or bone injuries; (4) other diseases affecting limb function and movement; (5) experience of high-intensity physical activities, muscle strength training, or other exercises for more than 15 min per time more than twice a week in the past 3 months; (6) participation in other forms of exercise during the study period.

### Randomization and allocation

Eligible participants were randomly assigned to either the DG or CG. The randomization sequence was computer generated and was concealed in sealed, opaque envelopes by a member of the research team not involved in recruitment. A therapist was responsible for sequentially opening the random assignment envelopes and allocating the participants accordingly.

## Outcome measures

The outcomes were measured by two physical therapists at 12 weeks and 24 weeks, which were expected to reflect the changes in the number of falls, fall injuries, functional mobility, and fear of falling.

### Primary outcome measures

The modified falls efficacy scale (MFES): Fall efficacy was measured using the MFES, a self-report scale measuring confidence in one's ability to avoid falling during the performance of activities in daily living. The MFES contains a total of 14 questions about basic daily activities, each of which is given 0 to 10 points (18). The MFES includes outdoor activities, which are not included in the Falls Efficacy Scale (FES) (19). The MFES was developed by Tinetti et al. as an instrument to measure fear of falling mainly among community-dwelling older individuals. The MFES seeks to determine the role of confidence and how confidence (or its opposite, fear) is implicated in falls (20).

Fall events: Participants and their families were asked to use a daily “fall calendar” diary to record all fall events (a fall is defined as “when you unintentionally land on the floor or the ground or fall and hit objects like stairs or pieces of furniture”), including the number of falls and fall injuries, and to indicate whether sought medical attention was sought.

### Secondary outcome measure

The Timed Up & Go (TUG) test: The TUG test requires the subject to rising from a chair, walk 3 m at a comfortable pace to a mark placed on the floor, turn around at the 3-m mark, walk back to the starting point, and return to a sitting position in the chair (21).

## Interventions

70 participants (21 men and 49 women) were randomly divided into the DG and CG Groups, each with 35 participants. The length of the intervention was 24 weeks. During the trial, each group participated in three 40-min educational sessions comprising the causes of falls and related risk factors, balanced self-assessment, selection of Auxiliary aid, and changes in the living environment designed to reduce the risk of falls. The participating therapist was required to have had 10 years of experience in traditional Chinese exercises and to be a skillful health care educator. The standard movements demonstrated by the therapist were recorded as videos and distributed to participants to guide their treatment.

### Ditangquan exercise group

Intervention methods in the DG consisted of 10 min of warm-up, 40 min of Ditangquan exercise, and 10 min of cooling down three times a week. We screened some movements from the Ditangquan exercise and designed a safe-landing program based on the older individuals' body conditions. The objective of the Ditangquan exercise is to teach older individuals how to fall in such a manner to reduce injury and alleviate impact severity. This consisted of three main parts:

(1) The first part involved protective techniques and practicing how to land safely when falling in different directions under controlled conditions so as to learn how to prevent injury in real-world situations. It is gradual training that reduces practice difficulty the elderly will not fall on the ground directly at the beginning. Furthermore, two professionals guided the elder's movements and ensured their safety on the spot during the exercise. The therapist placed gym mats on the ground and then instructed the participants to lower their center of gravity, roll their bodies, and increase the area of their body's contact with the ground to reduce impact load. The participants practiced the technique by falling onto the prepared gym mats while protecting themselves and making sure to avoid injury. It was explained that when one's body receives an impact, one must exhale to contract the muscles and constrict the rib cage, thus emptying the lungs of air. This helps to help protect the vital organs from damage.

(2) The second part was mainly related to muscle memory training. Participants were told to keep repeating the Ditangquan movements to protect themselves on the forward fall, sideward fall, and backward fall until a conditioning reflex was established. At this point, an older individual would have developed an adequate ability to protect herself or himself while falling. If this routine is practiced properly, the body should then instinctively exhale, relax, and allow the impact to spread through the body without injury.

(3) The third part mainly involved training in a simulated real-world environment. The participants had to instinctively and quickly make a series of movements to protect themselves when they suddenly felt themselves falling in different directions after an intended push by the therapist causing a loss of balance. These maneuvers were practiced 20 to 30 times in each direction. These exercises are so gentle and gradual that initially no participant falls directly to the ground. During the intervention, two professionals guide the participants' movements and ensure their safety. The essential movements of the Ditangquan exercises are illustrated in Supplementary Figures 1–3.

### The control group

Participants in the control group performed conventional exercises under the guidance of professionals, with the following structure for each session: 10 min of warm-up and stretching of the major lower limb muscles; 20 min of strength exercises; 20 min of aerobic activity (fast walking); and a 10-min cooling-down period, including gentle stretches and controlled breathing (22, 23).

## Sample size calculation and statistical analysis

PASS software (PASS 11, NCSS, LLC, Kaysville, UT) was used to estimate the sample size utilizing two independent sample means (α = 0.05, β = 0.10). The MFES was used as the primary efficacy outcome. According to a previous, similar clinical study (24), the average MFES score in the treatment group was 116.7, with a standard deviation of 12.1. The MFES score in the control group was 75.9, with a standard deviation of 23.0. In this study, the target sample size was 35 participants in each group, anticipating a maximal loss to follow-up of 15%.

All statistical analyses were performed using IBM SPSS version 24.0 software (SPSS Inc., Chicago, IL). The differences in primary and secondary outcomes between the two groups were tested by an intention-to-treat analysis (ITT). Missing data values were presumed to be missing at random, and calculations were completed using multiple inputs in the form of chained equations. We analyzed the differences between each endpoint before and after treatment. Data are presented as mean ± standard deviation (SD). All tests were conducted two-sided, and *P* ≤0.05 was considered statistically significant. The data that conformed to the normal distribution and variance homogeneity were analyzed by two independent sample *t*-test. The data that did not conform to the normal distribution were analyzed by the Wilcoxon rank-sum test. The numbers of falls and injuries were compared between the two groups by a chi-square test.

## Results

### Recruitment and baseline demographic data of the participants

**Figure 1:**
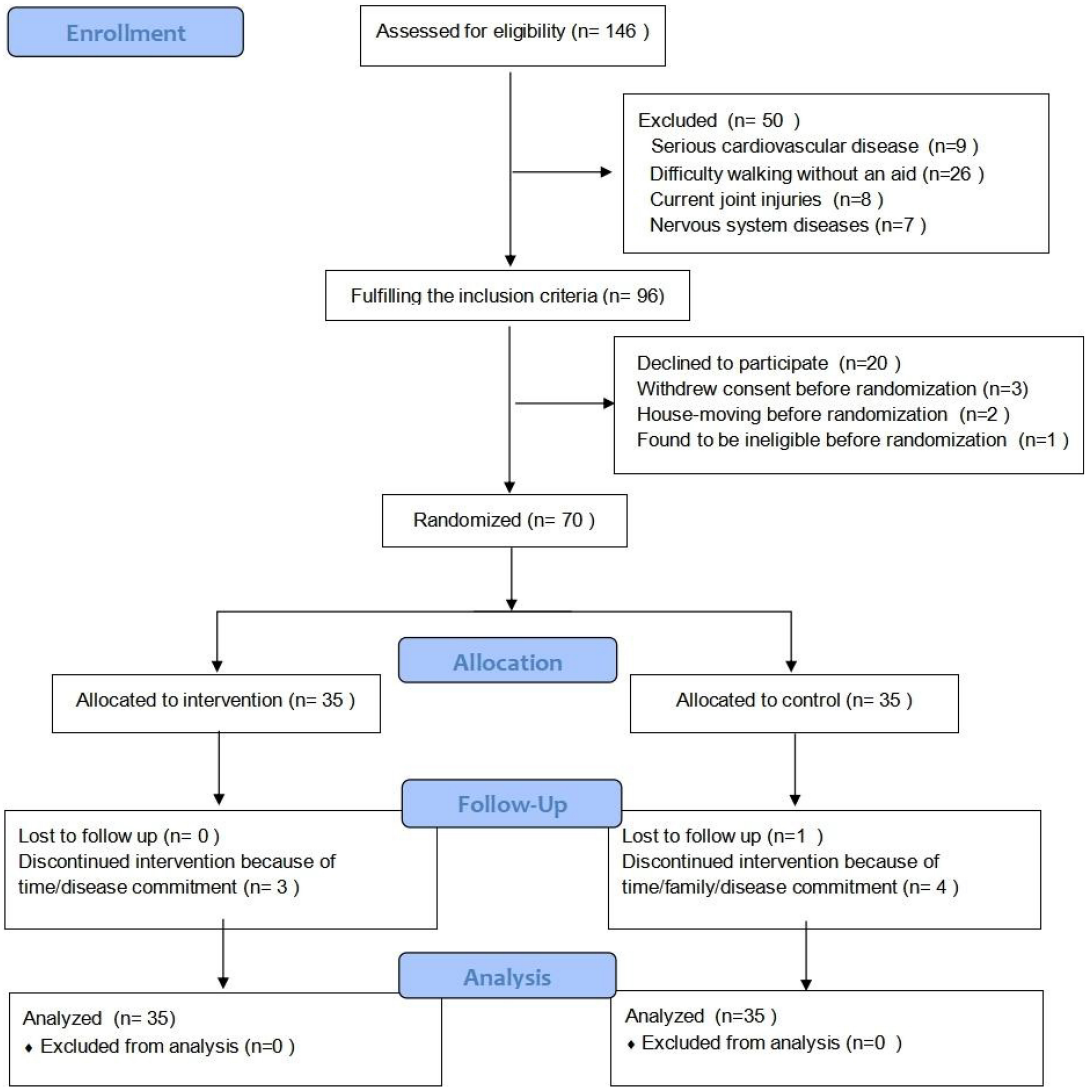
CONSORT diagram of the eligibility, exclusion and randomization scheme.

The flow diagram of the study and baseline data are presented in Figure 1 and Table 1, respectively. A total of 146 participants initially were recruited, and 76 were excluded. Specifically, 50 participants did not meet the inclusion criteria and 26 participants were unable to complete the entire study for various reasons. The remaining 70 participants met the eligibility criteria and signed the informed consent form. There were no differences between the two groups of participants insofar at the baseline as we were able to compare them. Of the 70 eligible participants, 62 completed the assessment at 24 weeks. A total of 3 participants in the DG and 5 participants in CG dropped out and contact was lost after several weeks of exercise. After 24 weeks, participants in the DG and CG completed 91.4 and 85.7%, respectively, of the total planned exercise sessions.

**Table 1:** Baseline demographic and clinical characteristics of the participants.

**Demographic characteristic**	**Ditangquan exercise group** **(*n* = 35)**	**Control group** **(*n* = 35)**	***P***
Age (years)	80.57 ± 8.93	77.89 ± 10.38	0.25
Gender			1
Male	10	11	
Female	25	24	
Height (cm)	160.95 ± 6.34	160.25 ± 7.70	0.329
Weight (kg)	57.83 ± 1.71	57.86 ± 1.90	0.991
BMI (kg/m^2^)	22.02 ± 0.49	22.59 ± 0.61	0.467
Education			0.647
Bachelor or above	4	8	
High school	10	8	
Middle school	10	9	
Primary school	11	10	
Number of falls			0.607
>2 times per year	12 (34%)	11 (31%)	
<2 times per year	23 (66%)	24 (69%)	
Fall injury	6	7	0.799
MFES	89.37 ± 31.88	80.51 ± 26.45	0.21
TUG test (second)	20.00 ± 10	18.07 ± 8.75	0.474

### Effects of Ditangquan exercise on falling events

The differences between the two groups in the number of fall events and injuries from baseline to 24 weeks are shown in Table 2. After the 24-week intervention, only one person in the DG fell, whereas eight participants in the CG fell (*P* = 0.028). In terms of fall-related injury, no participants in the DG were injured, whereas six participants in the CG experienced varying degrees of injury (e.g., wrist swelling, hip fracture, and spine injury). The DG was significantly different from the CG in terms of protecting the participants and preventing injuries (*P* = 0.025).

**Table 2:** Fall events between the two groups after the 24-week intervention.

	**DG**	**CG**	***P***
Fall	1	8	0.028
No fall	34	27	
Fall injury	0	6	0.025
No injury	35	29	

### Effects of Ditangquan exercise on the modified falls efficacy scale

**Figure 2:**
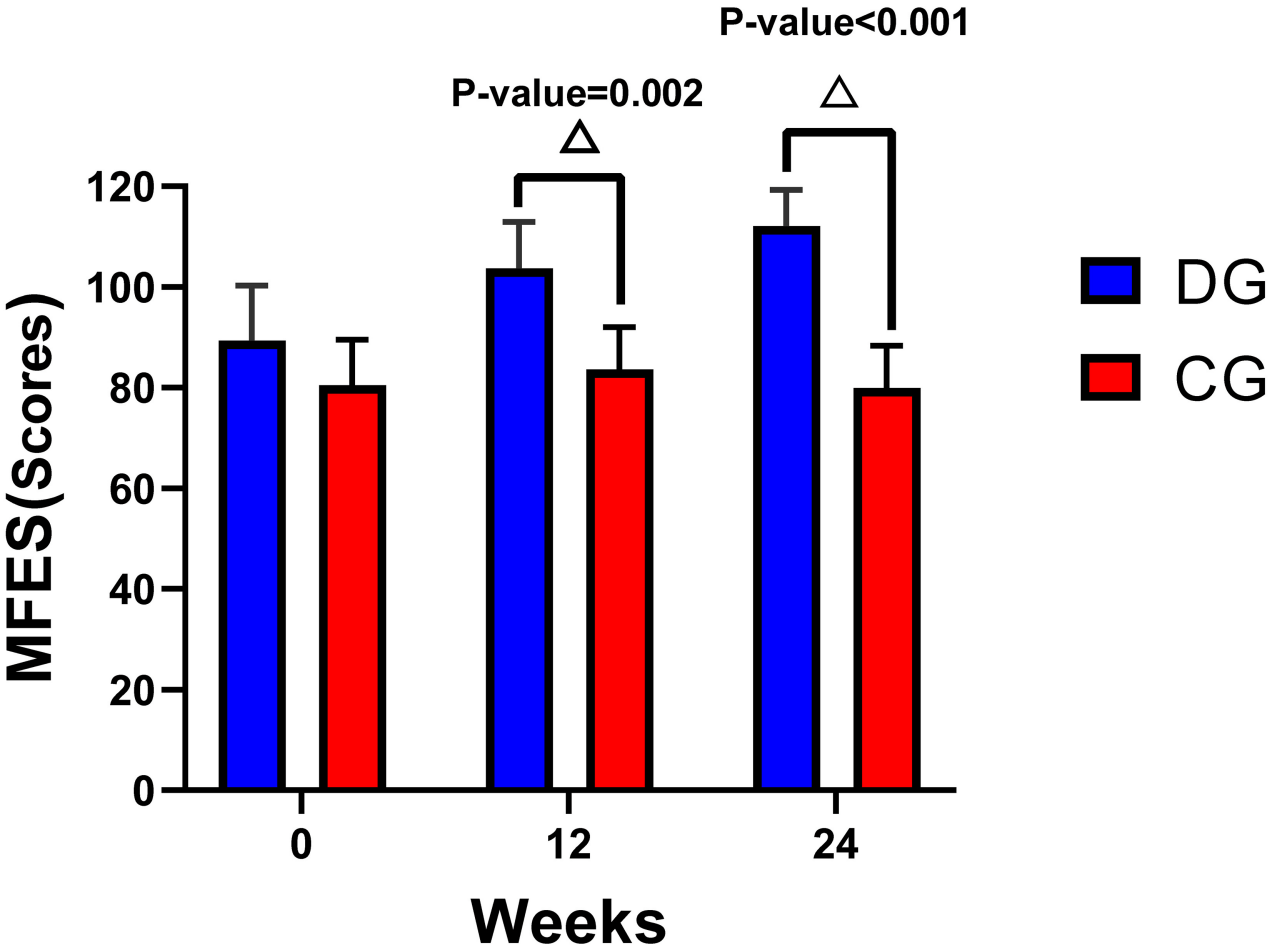
Changes in the MFES and TUG test from baseline to 12 weeks and 24 weeks. ^Δ^A significant difference between the two groups at 12 weeks and 24 weeks, *P* < 0.01.

Results showed that the MFES between the DG and CG differed significantly at 12 weeks (*P* = 0.002) and that the difference had become still greater at 24 weeks. This suggests that the Ditangquan exercises had a positive effect on improving the MFES in participants with reduced skeletal muscle function, and they became more effective as time went on. See Table 3 and Figure 2.

**Table 3:** Changes in the MFES and TUG test at 12 weeks and 24 weeks.

	**The difference in mean change** **(95% CI)**		**Between-group difference** **(95% CI)**	
**Outcomes**	**DG**	**CG**	**DG vs. CG**	***P***
**MFES**				
Week 12	14.29 (9.73, 18.84)*	3.14 (−1.41, 7.69)	20.00 (7.70, 32.30)^Δ^	0.002
Week 24	22.77 (16.66, 28.88)*	−0.54 (−6.66, 5.57)	32.17 (21.32, 43.02)^Δ^	<0.001
**TUG test**				
Week 12	3.13 (1.76, 4.50)*	−0.58 (−1.95, 0.79)	−2.50 (−5.57, 0.56)	0.107
Week 24	5.06 (3.45, 6.68)*	−1.09 (−2.70, 0.53)	−4.94 (−7.95,−1.93)^Δ^	0.002

^*^A significant change within the group from baseline to 12 weeks and 24 weeks, P < 0.05.

^Δ^A significant difference between the two groups at 12 weeks and 24 weeks, P < 0.01.

### Effects of Ditangquan exercise on the timed up and go test

**Figure 3:**
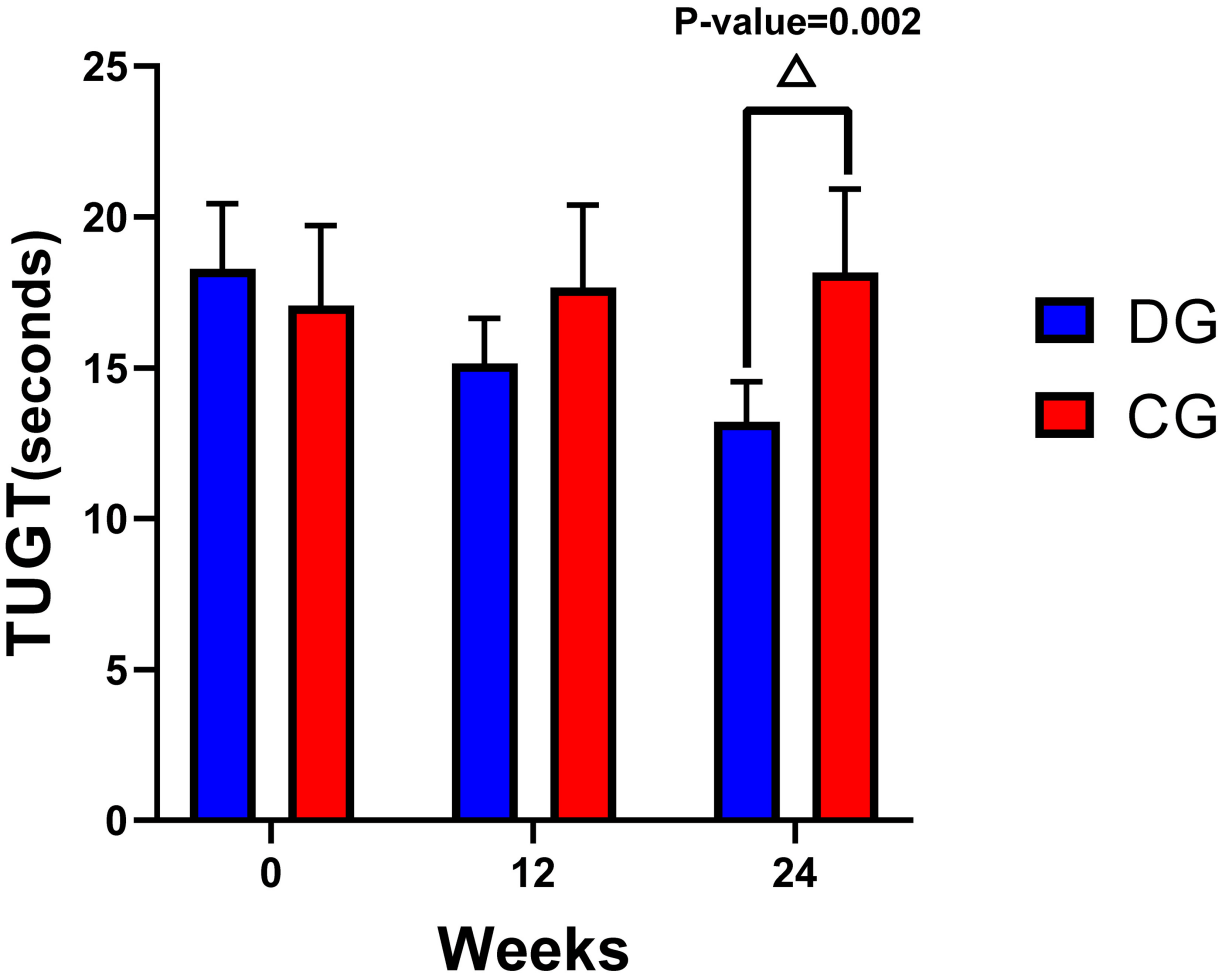
Changes in the MFES and TUG test from baseline to 12 weeks and 24 weeks. ^Δ^A significant difference between the two groups at 24 weeks, *P* < 0.01.

Results showed no significant difference in the TUG test between the two groups within 12 weeks of intervention (*P* = 0.107). At 24 weeks of intervention, the TUG test of the DG was significantly lower than that of the CG, and the difference was statistically significant (*P* = 0.002), as shown in Table 3 and Figure 3.

## Discussion

Falls are the second leading cause of unintentional injury and death among community-dwelling older individuals (25). They seriously affect the individual's psychological well-being while also reducing the quality of life. When an older individual falls, he or she must make some safe-landing movements that will reduce the force of impact, thereby reducing the risk of injury. Thus we designed an exercise program that would help to prevent falls and decrease the risk of fall-related injuries.

Ditangquan is a Chinese martial art that originated in the Shandong Province of China during the Song Dynasty (AD 960–1279). The major characteristic of this style is the ability to perform tumbles, falls, turns, flex elbows, squat, and then use those techniques for both offense and defense. Some techniques can help martial artists avoid injuries from falls. For example, when a martial artist's body receives an impact, he or she must exhale to constrict the rib cage, emptying the lungs of air to help protect the vital organs from damage. By practicing this properly and repeatedly, the individual can learn to exhale, relax, and instinctively allow the impact to spread throughout the body without sustaining any injury. Thus our exercise program as implemented in the DG was largely based on Ditangquan exercises and improved the physical condition of the participants. The DG exercise program mainly included tumbles, falls, turns, and rolls. We adopted the fundamental techniques of these movements to design a fall- and injury-proof exercise program. The core of the Ditangquan exercise is to land safely; it uses a unique protective technique to ensure safety and minimize the risks posed by falling (16). In the case of a fall, Ditangquan exercises cause the body to change its direction of motion and lower its center of gravity at the moment of touching the ground, thus reducing the reaction force of the body's impact and thus also the potential injury. Our results show that Ditangquan exercises based on safe-landing strategies effectively reduced the number of falls among older individuals, decreased injuries, improved dynamic balance, and increased the participants' confidence in their ability to prevent falls.

Older individuals are most likely to experience damage to the wrist and hip after falling, and it is difficult to make a full recovery from such injuries (26–29). When the wrist joint is subjected to external forces, 80% of the axial load borne by the distal radius may reach 3,100 to 3,200 N, which is enough to initiate a fracture (30–32). Several things can explain why the Ditangquan exercises help to prevent fall injuries in older individuals. First, the Ditangquan exercises employ rolling maneuvers to change the direction of the force, turning the potential force of gravity into kinetic energy and reducing the body's impact against the ground (33). Second, these techniques increase the contact area between the body and the ground. When a person falls, the larger the area of contact is, the less pressure is felt by the body, and the subsequent risk of injury decreases. Third, if one lowers one's center of gravity, the pressure on the hands and torso against the ground will be reduced. The population targeted in this study included older individuals with sarcopenia, and exercise is one of the interventions used to treat this condition (34–37). It not only effectively improves the patient's mobility but also delays the decline of skeletal muscle function due to aging (38, 39). Most of the Ditangquan movements can significantly improve the power and efficiency of both slow and fast muscle fibers, improve the contraction and coordination of skeletal muscle, and increase joint stability (40). As such, it increases the muscle strength of older individuals. In addition, these movements also involve speed, coordination, and responsive training, which improve balance and the ability to reduce falls.

The TUG test, which is widely used worldwide to assess balance among the elderly (41), was also used in this study. We found that there was no significant difference between the two groups at 12 weeks, but the TUG test scores in the DG were significantly decreased compared with those in the CG at 24 weeks. These findings indicate that if older individuals want to improve balance beyond that achieved with conventional exercise, a 24-week program of Ditangquan exercise is recommended.

In terms of the MFES, there was a significant difference between the two groups at 12 weeks, indicating that the participant's confidence in their ability to manage falling developed earlier than their improvement in balance. The individuals in the DG gradually felt an improvement in their functional mobility and coordination after Ditangquan exercise training, which enhanced their confidence in preventing falls. This increased self-confidence played a positive role in improving the ability of the participants to engage in physical activity. Owing to the decline in cognitive ability and mobility, older individuals correspondingly reduce the frequency of physical exercise, resulting in less movement overall and a reduced ability to move, eventually producing a vicious cycle. However, the Ditangquan exercises are based on a safe-landing strategy that can break this destructive cycle. Participants' minds focused on the process of training; they carried out both physical and mental exercises because traditional Chinese regimens also emphasize meditation (42). This psychological orientation effectively improves the participants' mental state (43) and decreases their level of anxiety and fear (44). Their improved confidence in preventing falls, gained through their improved mobility, enhances the older adults' interest in exercise, and this benefit stems from the integration of the Ditangquan exercises.

### Limitations of the study

Our study has some limitations. First, although statistically significant differences were found, the sample size was small. This problem may be alleviated in future studies seeking to confirm our findings. Second, ours is not a double-blind study because communication among residents in the same community cannot be avoided. This difficulty can be avoided by a cluster-randomized trial in the future. Third, our study focused mainly on observing the participants' motor function, questioning them regarding their confidence, and recording fall events. However, there is some evidence that the cognitive decline that is common among older adults and the occurrence of falls are correlated to some degree (45). Therefore more research is needed to observe the effects of Ditangquan exercise on the cognitive function of older individuals as well as their tendency to experience falls.

## Conclusion

Our findings indicate that Ditangquan exercise based on safe landing can effectively reduce the number of falls and fall injuries, improve the functional mobility and body coordination of older adults, and increase their confidence in their ability to prevent falls.

## Data availability statement

The raw data supporting the conclusions of this article will be made available by the authors, without undue reservation.

## Ethics statement

The studies involving human participants were reviewed and approved by Ethics Committee of Yueyang Hospital of Integrated Traditional Chinese and Western Medicine (No. 2018-013). The patients/participants provided their written informed consent to participate in this study.

## Author contributions

LF contributed to the conception and design of the study. Z-rL and Y-jM organized the database. X-cT performed the statistical analysis. Z-rL wrote the first draft of the manuscript. C-yG, S-tL, R-rZ, and J-xW wrote sections of the manuscript. All authors contributed to manuscript revision, read, and approved the submitted version.

## Funding

This work was supported by the Three-Year Action Plan for the Development of TCM in Shanghai–Highland Construction for International Standardization of TCM [No. ZY (2021–2023)-0212].

## Conflict of interest

The authors declare that the research was conducted in the absence of any commercial or financial relationships that could be construed as a potential conflict of interest.

## Publisher's note

All claims expressed in this article are solely those of the authors and do not necessarily represent those of their affiliated organizations, or those of the publisher, the editors and the reviewers. Any product that may be evaluated in this article, or claim that may be made by its manufacturer, is not guaranteed or endorsed by the publisher.
