# Supplementary material for: Ditangquan exercises based on safe-landing strategies prevent falls and injury among older individuals with sarcopenia
Source: Front Med (Lausanne). 2022 Aug 16;9:936314. doi: 10.3389/fmed.2022.936314 (PMC9424729; doi:10.3389/fmed.2022.936314)

All movements are selected from Ditangquan exercise and revised according to the physical conditions of the elderly.

1. Forward fall safe-landing training

Tips of movements：

(1)Practice self-protection movements when falling forward on a cushion. Extend your arms as far forward as possible, bend your knees, and reduce the distance between your hands and the ground. After touching the ground with both hands, quickly flex the elbow joint, put it in front of the chest to protect the lung, heart, and rib, breath out at the same time, raise your head and extend your back. Let the hands, elbows, chest, abdomen, and thighs land on the ground in turn during the fall.

(2)After completing the safe landing movement, whether the range of motion of wrist and elbow joints is limited and whether there will be a pain in the chest and abdomen.

(3)Repeat the training on the cushion until the practitioner’s whole body can coordinate and skillfully complete the movement. Then simulating a real-world environment on the cushion, the practitioner need quickly completes the self-protection action when the therapist suddenly gives a push to make the practitioner loss of his balance.

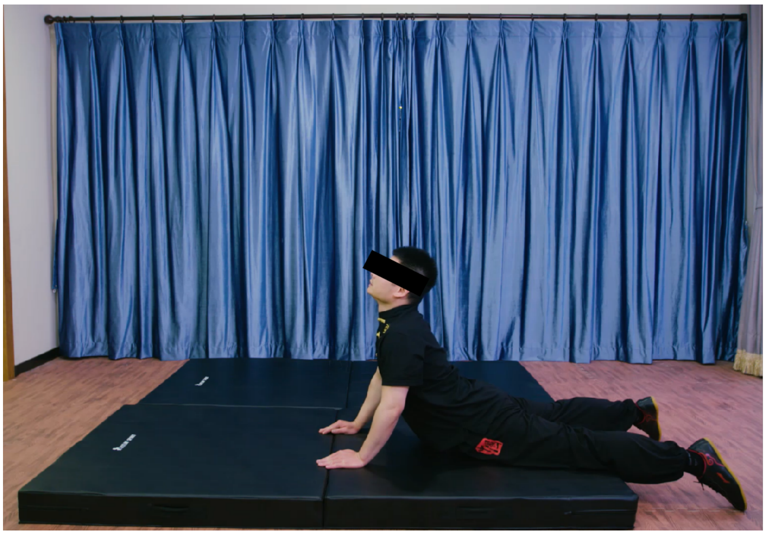

Supplementary Figure 1. Forward fall safe-landing training

2. Sideward fall safe-landing training

Tips of movements：

(1)Practice self-protection movements when falling sideward on a cushion. Quickly split the legs to reduce the height of the body's center of gravity. meanwhile, turn the upper body to the side, bend the elbow and lift it up with one hand to protect the head, and put the other hand on the side of the chest to protect the ribs. The hip joint abduction does not touch the ground. After completing the movement, check whether there will be a pain in the wrist, elbow, hip movement, chest, and abdomen or breathing.

(2)Repeat practice the sideward rotation, protect head, chest, and hip abduction on the cushion until the practitioner finishes the movement skillfully.

(3)Simulated real-world environment on the cushion, the practitioner need quickly completes the self-protection action when the therapist suddenly gives a side push to make the practitioner lose his balance.

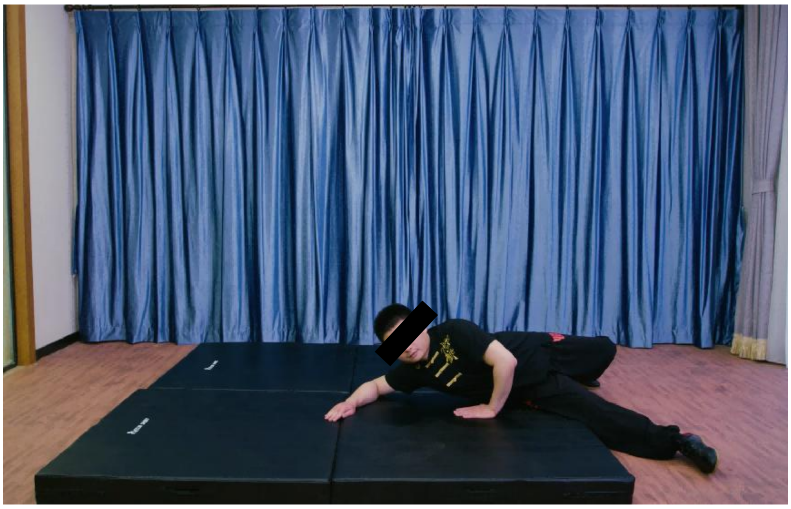

Supplementary Figure 2. Sideward fall safe-landing training

3. Backward fall safe-landing training

Tips of movements：

(1)Practice self-protection movements when falling backward on a cushion. Quickly bend your knees to reduce the height of your body's center of gravity. When you fall back, bend your neck to protect your head. Extend your hands to both sides of the body, touch the ground first, and then roll over your forearm, upper arm, waist, and back, in turn, to avoid landing on your buttock. Meanwhile, flex the abdomen and lower limbs and roll like a ball. After completing the action, check whether there will be a pain in the wrist, elbow, and back.

(2)Repeat neck flexion and back rolling on the cushsion until the practitioner finishes the movement skillfully.

(3)Simulated real-world environment on the cushion, the practitioner need quickly completes the self-protection action when the therapist suddenly gives a back push to make the practitioner lose his balance.

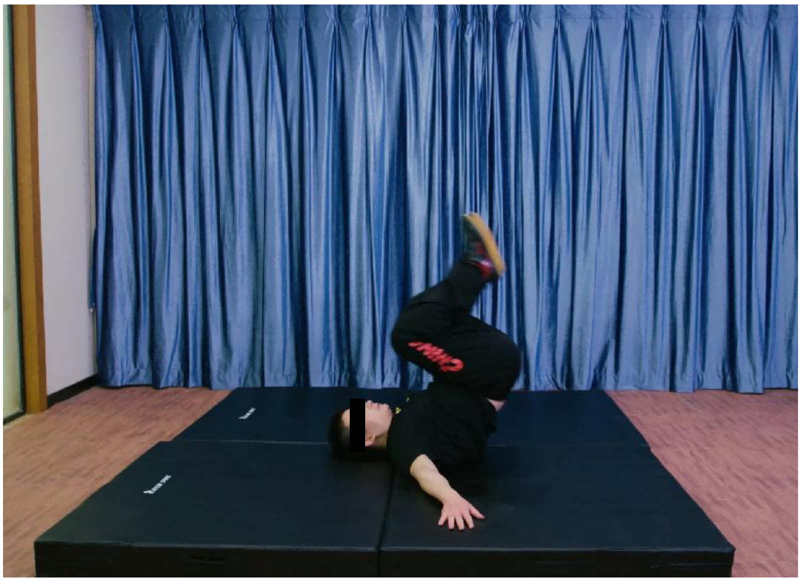

Supplementary Figure 3. Backward fall safe-landing training

The photos of older individuals practice Ditangquan exercise

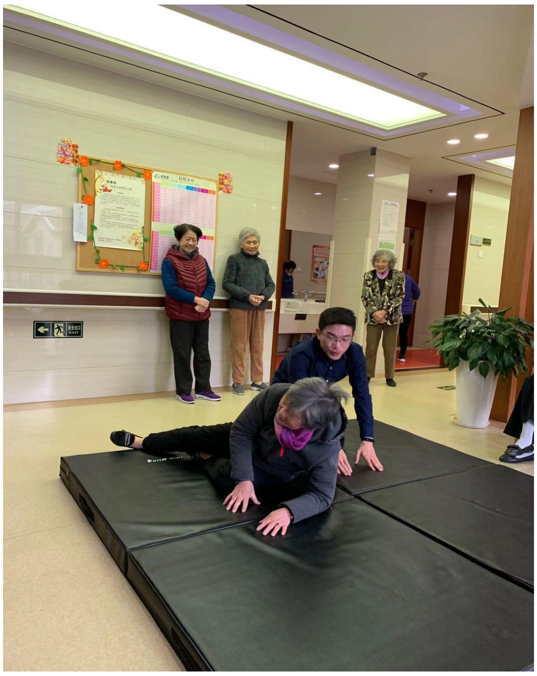

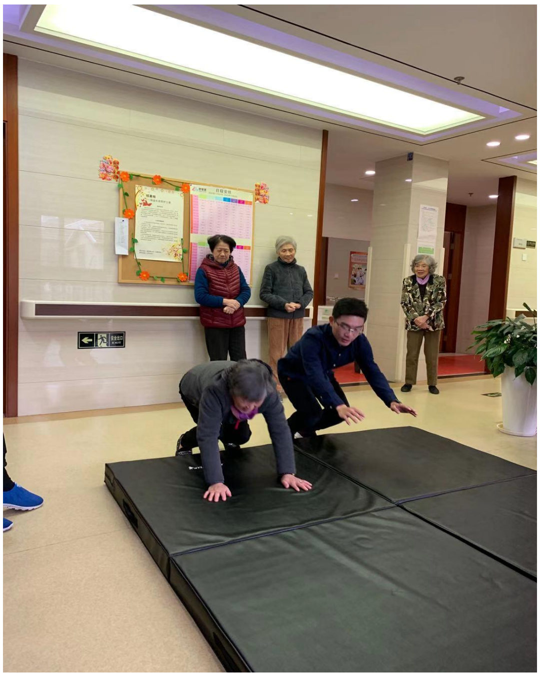

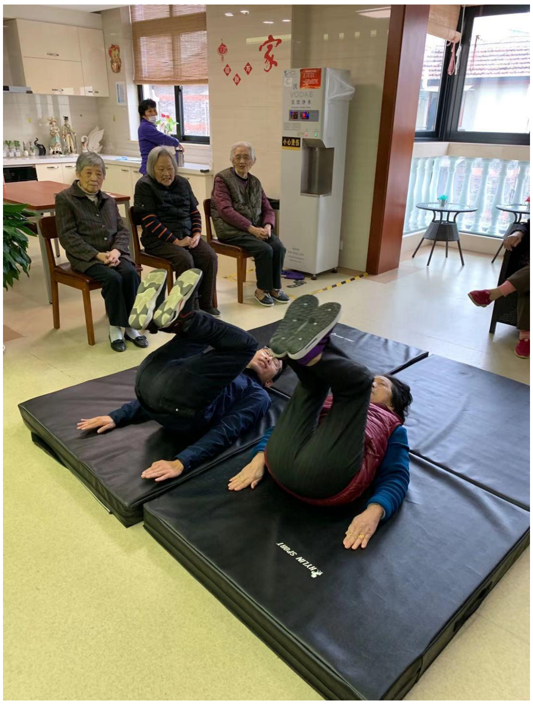

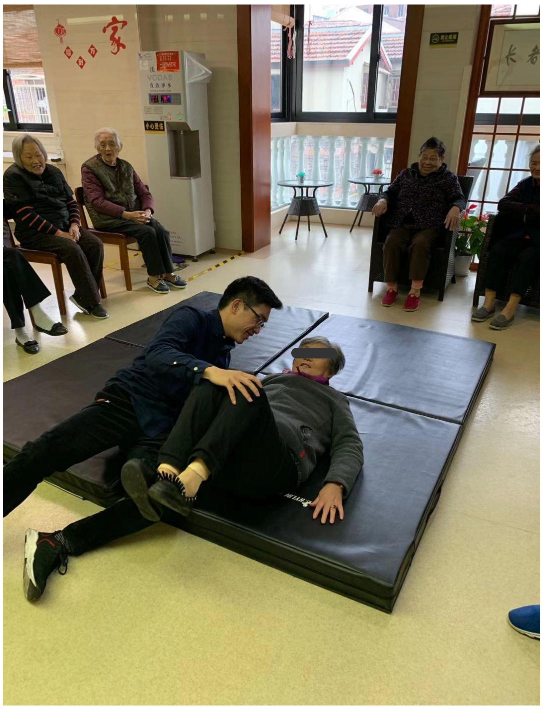

Supplement: Supplementary file 1 [file Data_Sheet_1.pdf]
